# Obstructive Sleep Apnea Syndrome in Children with 22q11.2 Deletion Syndrome after Operative Intervention for Velopharyngeal Insufficiency

**DOI:** 10.3389/fped.2014.00084

**Published:** 2014-08-11

**Authors:** David Jeffrey Crockett, Steven L. Goudy, Sivakumar Chinnadurai, Christopher Todd Wootten

**Affiliations:** ^1^Department of Otolaryngology, Vanderbilt University Medical Center, Nashville, TN, USA

**Keywords:** obstructive sleep apnea, velopharyngeal insufficiency, velopharyngeal dysfunction, 22q11.2 deletion syndrome, velocardiofacial syndrome, DiGeorge syndrome, pharyngeal flap, Furlow palatoplasty

## Abstract

**Introduction:** Surgical treatment of velopharyngeal insufficiency (VPI) in 22q11.2 deletion syndrome is often warranted. In this patient population, VPI is characterized by poor palatal elevation and muscular hypotonia with an intact palate. We hypothesize that 22q11.2 deletion patients are at greater risk of obstructive sleep apnea (OSA) after surgical correction of VPI, due, in part, to their functional hypotonia, large velopharyngeal gap size, and the need to surgically obstruct the velopharynx.

**Methods:** We performed a retrospective analysis of patients with 22q11.2 deletion syndrome treated at a tertiary pediatric hospital between the years of 2002 and 2012. The incidence of VPI, need for surgery, post-operative polysomnogram, post-operative VPI assessment, and OSA treatments were evaluated.

**Results:** Forty-three patients (18 males, 25 females, ages 1–14 years) fitting the inclusion criteria were identified. Twenty-eight patients were evaluated by speech pathology due to hypernasality. Twenty-one patients had insufficient velopharyngeal function and required surgery. Fifteen underwent pharyngeal flap surgery, three underwent sphincter pharyngoplasty, two underwent Furlow palatoplasty, and one underwent combined sphincter pharyngoplasty with Furlow palatoplasty. Of these, eight had post-operative snoring. Six of these underwent polysomnography (five underwent pharyngeal flap surgeries and one underwent sphincter pharyngoplasty). Four patients were found to have OSA based on the results of the polysomnography (average apnea/hypopnea index of 4.9 events/h, median = 5.1, SD = 2.1). Two required continuous positive airway pressure (CPAP) due to moderate OSA.

**Conclusion:** Surgery is often necessary to correct VPI in patients with 22q11.2 deletion syndrome. Monitoring for OSA should be considered after surgical correction of VPI due to a high occurrence in this population. Furthermore, families should be counseled of the risk of OSA after surgery and the potential need for treatment with CPAP.

## Introduction

The 22q11.2 deletion syndrome is the most frequent human microdeletion syndrome (1). It has a frequency of 1:6000–1:2000 (2, 3). Significant phenotypic heterogeneity is found among affected individuals. Anatomic and functional concerns include characteristic facies, congenital heart disease, immune disorders, hypocalcemia, hearing loss, cleft palate, hypotonia, feeding difficulties, developmental delay, psychiatric issues, speech delay, and velopharyngeal insufficiency (VPI) (4–6).

**Figure 1:**
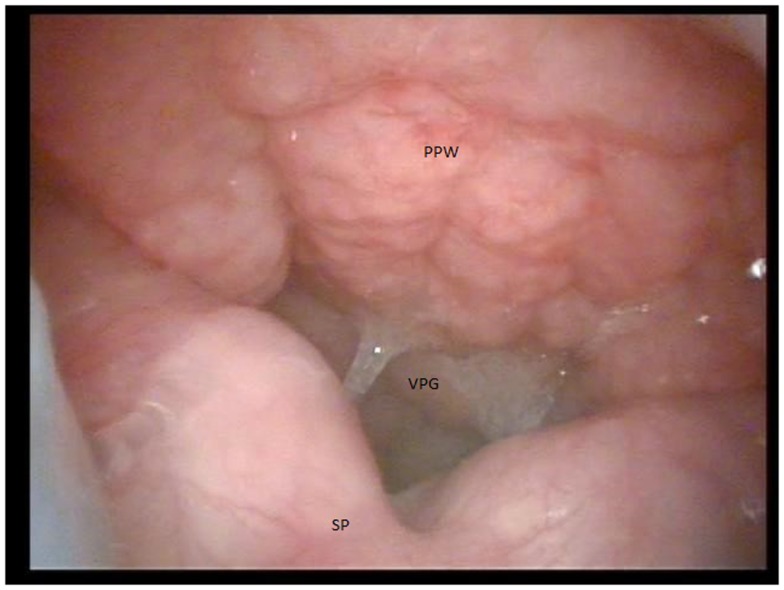
**Image of a persistent velopharyngeal gap causing velopharyngeal insufficiency in a patient with a submucous cleft palate undergoing video nasopharyngeal endoscopy (PPW = posterior pharyngeal wall, VPG = velopharyngeal gap, SP = soft palate)**.

The incidence of VPI in patients with 22q11.2 deletion syndrome ranges from 27% of patients to 80% (7, 8). Poor functioning of the velopharyngeal mechanism may be due to various anatomic and functional reasons. Overt cleft palate and submucous cleft palate are infrequently identified in patients with this syndrome and are known to be associated with VPI (Figure 1) (9). Generalized muscular hypotonia, also, often affects the muscles of the palate, contributing to a poor velopharyngeal mechanism.

The VPI associated with 22q11.2 deletion syndrome is known to be more difficult to treat, and studies have demonstrated inferior surgical outcomes when patients with this syndrome are compared to non-syndromic patients (10, 11). However, surgical treatment of VPI in these patients is often warranted to correct their resonance disorder. Surgical options to correct VPI include posterior pharyngeal augmentation, palatoplasty (double opposing *z*-plasty and intravelar muscular repositioning), sphincter pharyngoplasty, pharyngeal flap pharyngoplasty, and combinations of these procedures. Each of these procedures narrow the velopharyngeal port and thus predispose patients to obstructive sleep apnea (OSA) (12).

In recent years, OSA occurrence has been increasingly acknowledged as a frequent and significant complication after the correction of VPI. Studies have demonstrated a significant risk of OSA within non-syndromic patients undergoing velopharyngeal surgery for VPI (13–15). The incidence of OSA in the 22q11.2 deletion syndrome population is not well described. Decreased upper airway tone with pharyngeal and palatal muscular hypotonia can contribute to OSA (16). Additionally, the high rate of congenital cardiac malformations in this population may create a physiology, which is not tolerant for the potential hypoxia associated with OSA (17).

The risk of OSA in the 22q11.2 deletion syndrome population after surgery for VPI is also poorly described. The aim of this study is to evaluate patients with 22q11.2 deletion syndrome and VPI necessitating surgical intervention in order to determine the risk of OSA. We hypothesize that these patients are at greater risk of OSA post-operatively, due, in part, to their functional hypotonia and the need to surgically obstruct the velopharynx.

## Materials and Methods

Approval for the study was obtained by an institutional review board at Vanderbilt University. A retrospective review of patients with 22q11.2 deletion syndrome treated at the tertiary pediatric hospital associated with Vanderbilt University was performed between the years of 2002 and 2012. Inclusion criteria included all patients with 22q11.2 deletion syndrome under the age of 18 years at treatment as identified through an electronic medical record search. Genetic evaluation in conjunction with the results of the fluorescent *in situ* hybridization test was identified in order to confirm the diagnosis of 22q11.2 deletion syndrome in all patients included in the study.

**Figure 2:**
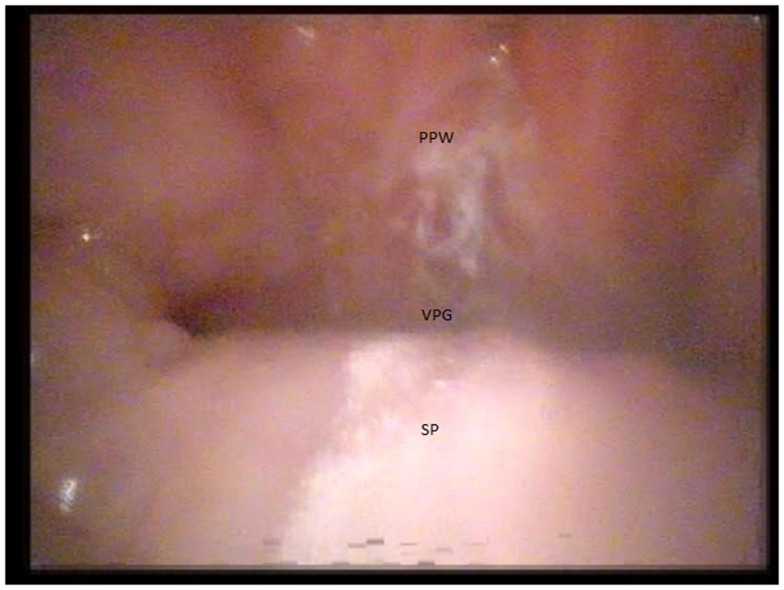
**Image of a persistent large velopharyngeal gap causing velopharyngeal insufficiency in a patient with 22q11.2 deletion syndrome undergoing video nasopharyngeal endoscopy (PPW = posterior pharyngeal wall, VPG = velopharyngeal gap, SP = soft palate)**.

The surgical treatments were also assessed to ensure that they were performed between the years included in the review. Patients were only referred for surgical treatment after aggressive speech therapy. All patients were evaluated by two speech-language pathologists and a surgeon experienced in the treatment of VPI prior to any recommendation for surgery. All patients undergo video nasopharyngeal endoscopy to evaluate the velopharyngeal function, closure pattern, and velopharyngeal gap prior to surgery (Figure 2). The operative procedures performed for each patient were decided based on the expertise of the treating surgeon using the results of the video nasopharyngeal endoscopy as a guide. Patients with large velopharyngeal gaps and those with an adynamic velopharyngeal mechanism underwent a pharyngeal flap procedure. Those with lateral gaps or “bowtie” closure patterns underwent a sphincter pharyngoplasty. Furlow palatoplasty (18) was reserved for patients with small gaps with a dynamic velopharyngeal mechanism and for those with a submucous cleft palate. The combination of Furlow palatoplasty and sphincter pharyngoplasty was recommended in patients with a moderate velopharyngeal gap.

The incidence of VPI, need for surgery, post-operative polysomnogram (PSG), post-operative VPI assessment, and OSA treatments were specifically evaluated. The primary outcome measure was incidence and severity of OSA, as confirmed by polysomnography, after velopharyngeal surgery. Sleep apnea was diagnosed when the apnea/hypopnea index (AHI) on polysomnography was >1 event/h (19). An increased AHI was considered to be consistent with an increase in the severity of the sleep apnea. Severity of sleep apnea was categorized as mild (AHI >1 event/h to 5 events/h), moderate (AHI between 5 and 10 events/h), or severe (AHI >10 events/h).

## Results

Forty-three patients (18 males, 25 females, ages 1–14 years) fitting the inclusion criteria were identified. Twenty-eight of the 43 patients were evaluated by the speech-language pathology team due to concern for hypernasality. Seven of these 28 patients had sufficient velopharyngeal closure for normal speech and were treated with speech therapy. Twenty-one of the 28 patients evaluated by the speech-language pathology team had insufficient velopharyngeal function and were subsequently referred for surgery. Fifteen of the 21 patients underwent pharyngeal flap surgery, 3 of the 21 underwent sphincter pharyngoplasty, 2 of the 21 underwent Furlow palatoplasty, and 1 of the 21 underwent combined sphincter pharyngoplasty with Furlow palatoplasty (Table 1).

**Table 1:** **Patient characteristics of those included in the study with the corresponding values**.

Patient Characteristics	Value
Total patients with 22q11.2 deletion syndrome	43
Female (% of total)	25 (58)
Male (% of total)	18 (42)
Patients with VPI requiring surgery (% of total)	21 (49)
Pharyngeal flap (% of pts requiring surgery)	15 (71)
Sphincter pharyngoplasty (% of pts requiring surgery)	3 (14)
Furlow palatoplasty (% of pts requiring surgery)	2 (10)
Combination Furlow and Sphincter (% of pts requiring surgery)	1 (5)
Patients diagnosed with OSA based on PSG (% of pts requiring surgery)	4 (19)
Patients requiring CPAP (% of pts requiring surgery)	2 (10)

Of the 21 patients who required velopharyngeal surgery, 5 had a submucous cleft palate and 1 had a cleft of the secondary palate. The remainder did not have an identifiable anatomic difference. All patients demonstrated improvement in their post-operative perceptual speech evaluation by the speech-language pathology team. However, two patients had persistent VPI to the extent that further surgery was recommended. One of these underwent a revision of their pharyngeal flap and the other a revision of their sphincter pharyngoplasty. Adequate improvement was noted by the speech-language pathology team after the revision was performed in the patient with the pharyngeal flap. However, a second revision was necessary for the patient with the sphincter pharyngoplasty. After the second revision, the patient had sufficient velopharyngeal closure for speech, as assessed by the speech-language pathology team.

Post-operatively, eight patients began snoring. Six of these eventually underwent polysomnography due to the severity of the snoring and a clinical suspicion for OSA (five had pharyngeal flap surgeries and one underwent a sphincter pharyngoplasty, ages 3–6 years). The sleep studies were performed at an average of 2.6 years after the surgery for VPI (median = 1.7, SD = 2.3, range of 6 months–6 years). The average AHI of all those undergoing post-operative polysomnography was 3.6 events/h (median = 3.3, SD = 2.6). The results of the polysomnography demonstrated OSA in four patients (average AHI of 4.9 events/h, median = 5.1, SD = 2.1). Two patients were placed on continuous positive airway pressure (CPAP) due to moderate OSA with an average AHI of 6.6 events/h. Both of these patients had already undergone further surgical treatment for sleep disordered breathing prior to their PSG. One patient underwent a tonsillectomy and the other underwent a midline posterior glossectomy with a lingual tonsillectomy (as an adenotonsillectomy had already been performed earlier in life due to concern for sleep disordered breathing). The results of the remaining two patients demonstrated mild OSA with an average AHI of 3.3 events/h and both were treated conservatively (monitoring and/or a nasal steroid spray with montelukast). Table 2 lists the treatments and results of all the patients who underwent PSGs.

**Table 2:** **Demographics of the patients who underwent polysomnography, including the surgery performed for their VPI, pre and post-polysomnography treatments of OSA, and AHI results of the PSG**.

Age (in years)	Sex	VPI Surgery	Pre-PSG OSA surgery	Post-OP AHI	OSA treatment
3	F	Pharyngeal flap	Adenoidectomy	1.0 events/h	Conservative
4	F	Sphincter pharyngoplasty	None	4.1 events/h	Conservative
4	M	Pharyngeal flap	T&A, MPG, LT	6.1 events/h	CPAP
5	M	Pharyngeal flap	Tonsillectomy	7.0 events/h	CPAP
6	M	Pharyngeal flap	None	1.0 events/h	Conservative
6	F	Pharyngeal flap	T&A	2.4 events/h	Conservative

*PSG = polysomnography, AHI = apnea/hypopnea index, OSA = obstructive sleep apnea, T&A = tonsillectomy and adenoidectomy, MPG = midline posterior glossectomy, LT = lingual tonsillectomy*.

Of note, one of the patients on CPAP therapy began to have improved sleep symptoms after 3 years and a repeat PSG was performed. The study demonstrated an AHI of 1.8 events/h and he was able to discontinue CPAP therapy. Only one patient with OSA confirmed by polysomnography underwent a PSG prior to velopharyngeal surgery. The initial study was performed 1 year prior to surgery on the velopharynx, but after an adenotonsillectomy for symptoms consistent with sleep disordered breathing. The study demonstrated an AHI of 0.2 events/h. This patient subsequently underwent a repeat PSG 1 year after surgery, which demonstrated mild OSA (AHI of 2.4 events/h).

## Discussion

This study demonstrates that OSA is a possible postoperative complication in patients with 22q11.2 deletion syndrome who undergo surgery for VPI. OSA was confirmed with polysomnography in 4 of the 21 patients (19%) who required surgery. Fortunately, those identified with OSA were all in the mild to moderate range (AHI <10 events/h). Only two patients were placed on CPAP due to concern for the severity of their OSA. One of these patients was able to discontinue CPAP after 3 years due to improvement in their PSG. The remaining patients in the study did not undergo polysomnography and thus we cannot confirm or deny OSA in this population. This group of patients either did not snore and/or the clinical suspicion for OSA was not present. Of note, only one patient with confirmed OSA underwent a sleep study prior to proceeding with surgery on their velopharyngeal port, making the data difficult to interpret.

Recent studies have started to take into account the concern for OSA when performing velopharyngeal surgery, especially on syndromic populations. Rottgers et al. (17) proposed an algorithm for the application of the Furlow palatoplasty in treating VPI in patients with 22q11.2 deletion syndrome. Essentially, the authors propose that all patients with an overt cleft or a submucous cleft palate with a kinetic palate should undergo a Furlow palatoplasty (pharyngeal flaps were performed for revisions and akinetic palates). One of the primary reasons for this proposal, according to the authors, is that Furlow palatoplasty can be effective in select populations and is less likely to cause post-operative OSA. Using this method, the authors did not have any patients present with symptoms of OSA, although polysomnography data are not included. They found this algorithm to be successful in the treatment of VPI in many patients with 22q11.2 deletion syndrome, although the potential for secondary pharyngoplasty must be considered.

Similarly, recent studies have also demonstrated the effectiveness of performing a pharyngeal flap or sphincter pharyngoplasty in patients with 22q11.2 deletion syndrome and VPI (20–22). Spruijt et al. (21) performed a systematic review in search of the optimal surgical treatment for velopharyngeal dysfunction in 22q11.2 deletion syndrome. Based on their review, they concluded that a grade C recommendation could be made to perform a pharyngoplasty directly in order to minimize the morbidity of further revision surgery that is more likely to occur when a palatoplasty alone is performed. Interestingly, in that review, only 1% of the 525 patients with 22q11.2 deletion syndrome were noted to have OSA after surgery for VPI. In all of these studies, the diagnosis and incidence of OSA is often minimized and based solely on clinical symptomatology and not polysomnography. Several studies of pediatric OSA have confirmed that clinical symptoms are poor predictors of OSA demonstrated on polysomnography (23–25).

Though our study is a retrospective review, it does demonstrate that within this syndromic population, a risk of OSA exists following operative repair of VPI, and the rate of OSA is higher than previously reported. Clinicians should have a low threshold to perform polysomnography on patients with 22q11.2 deletion syndrome following VPI operations. While the ideal interval is unknown, waiting 6 months postoperatively may allow for resolution of temporary postoperative edema. Assessment of postoperative OSA is especially important in the 22q11.2 deletion syndrome population as there is a high rate of comorbidities, such as cognitive delays and cardiac anomalies, which would be negatively impacted by fragmented sleep, catecholamine dysregulation, and hypoxia/hypercarbia. Further studies are necessary in order to quantify the risk of postoperative (and preoperative) OSA, determine its severity, and identify variables to improve outcomes.

## Conclusion

Surgery is often used to treat VPI in patients with 22q11.2 deletion syndrome. OSA after surgery for VPI in this population is a known complication. Based on this retrospective review, monitoring for OSA should be considered before and after surgery due to its known occurrence and the severity of morbidity that may be associated with it, especially in these syndromic patients. Furthermore, families should be counseled of this risk after surgery and the potential need for CPAP treatment. Additional prospective studies with pre- and post-surgical polysomnography data are needed to further explore the complication of OSA after surgery on the velopharynx.

## Author Contributions

All authors (David Jeffrey Crockett, Steven L. Goudy, Sivakumar Chinnadurai, Christopher Todd Wootten) provided substantial contributions to the acquisition, analysis, and interpretation of the data included in this study. All authors contributed to drafting and revising the content of this manuscript. Final approval for the publication of this work was noted by all of the authors, including an agreement to be accountable for all aspects of the work. David Jeffrey Crockett and Christopher Todd Wootten contributed to the conception and design of the study.

## Conflict of Interest Statement

The authors declare that the research was conducted in the absence of any commercial or financial relationships that could be construed as a potential conflict of interest.
